# BNT162b2 vaccination effectively prevents the rapid rise of SARS-CoV-2 variant B.1.1.7 in high-risk populations in Israel

**DOI:** 10.1016/j.xcrm.2021.100264

**Published:** 2021-04-18

**Authors:** Ariel Munitz, Matan Yechezkel, Yoav Dickstein, Dan Yamin, Motti Gerlic

**Affiliations:** 1 Department of Clinical Microbiology and Immunology, Faculty of Medicine, Tel Aviv University, Tel Aviv 6997801, Israel; 2 Electra-TAU laboratory, Omer, Israel; 3 Laboratory for Epidemic Modeling and Analysis, Department of Industrial Engineering, Faculty of Engineering, Tel Aviv University, Tel Aviv 6997801, Israel; 4 Center for Combatting Pandemics, Tel Aviv University, Tel Aviv 6997801, Israel

**Keywords:** SARS-CoV-2, COVID-19, surveillance, vaccination, viral infection, variant, transmission, B.1.1.7, vaccine, BNT162b2

## Abstract

Since the emergence of the SARS-CoV-2 pandemic, various genetic variants have been described. The B.1.1.7 variant, which emerged in England during December 2020, is associated with increased infectivity. Therefore, its pattern of spread is of great importance. The Israeli government established three national programs: massive RT-PCR testing, focused surveillance in nursing homes, and robust prioritized vaccination with BNT162b2. To define the impact of the aforementioned programs, we analyze data from ∼300,000 RT-PCR samples collected from December 6, 2020, to February 10, 2021. We reveal that the B.1.1.7 is 45% (95% confidence interval [CI]: 20%–60%) more transmissible than the wild-type strain and has become the dominant strain in Israel within 3.5 weeks. Despite the rapid increase in viral spread, focused RT-PCR testing and prioritized vaccination programs are capable of preventing the spread of the B.1.1.7 variant in the elderly. Therefore, proactive surveillance programs, combined with prioritized vaccination, are achievable and can reduce severe illness and subsequent death.

## Highlights

The B.1.1.7 variant is 45% more transmissible than the wild-type strain in Israel
Active surveillance markedly reduces the transmission of B.1.1.7 in nursing homes
Prioritized vaccination prevents B.1.1.7-associated infections in the elderly
Proactive surveillance, combined with prioritized vaccination, is achievable

## Graphical abstract

**Figure:**
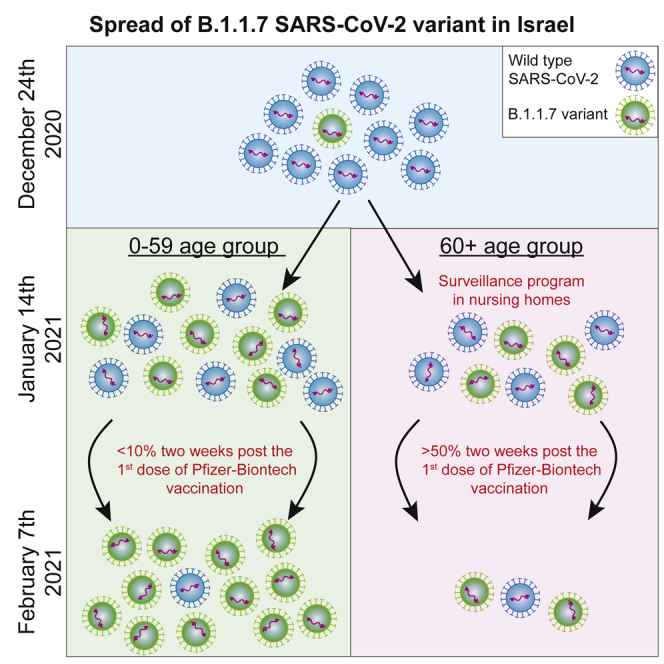

## Teaser

Munitz et al. demonstrate that, despite a 45% increased transmission of the B.1.1.7 SARS-CoV-2 variant, the Israeli vaccination program curtails its spread in elderly populations. Furthermore, pro-active surveillance programs likely enable the containment of viral spread in nursing homes. Thus, combined programs are achievable, reduce severe illness, and subsequent death.

## Introduction

In December 2020, a distinct, phylogenetic cluster of SARS-CoV-2 was identified in London, UK, as well as in southeast and east England.^1^ Since the emergence of this variant (now termed variant B.1.1.7), it has been shown to display a number of mutations that have been postulated to affect its infectivity and, thus, its spread in the community.^2^ Variant B.1.1.7 is defined by 17 mutations; among which, several are located in the spike protein that mediates SARS-CoV-2 attachment and entry into human epithelial cells.^3^^,^^4^ At least two mutations have been suggested to have biological significance. Mutation N501Y has been shown to enhance binding affinity to human angiotensin-converting enzyme 2 (ACE2),^3^^,^^4^ whereas the 69/70 deletion was shown to increase the infectivity of B.1.1.7 *in vitro* in a pseudovirus infection model.^5^ Another mutation, P681H, which is near the furin-cleavage site and, thus, may enhance the cleavage, has been suggested to affect infection and transmission.^6^ Nevertheless, increased transmissibility for the B.1.1.7 in real life, outside of the UK, is still unclear. This is important specifically because additional variants, including the California variants B.1.526 and B.1.138, which do not contain the N501Y mutation, were also shown to be more transmissible.^3^ Preliminary evidence from epidemiological studies in England estimated that B.1.1.7 is 43%–82% more transmissible^7^ and is, thus, associated with an increase in the effective reproduction number (*R*_*t*_) by a factor of 1.4–1.8.^8^ Despite these data, it has not yet been established whether the B.1.1.7 is indeed more infective, and if so, what the magnitude of that change is, especially outside of England. Furthermore, it has not yet been established whether and how quickly the B.1.1.7 variant can overtake the non-N501Y SARS-CoV-2 strain in community settings. Finally, interventions that may slow the spread of the B.1.1.7 variant remain to be determined.

During the COVID-19 pandemic, Israel established three noteworthy programs. The first, is a high-throughput, national RT-PCR testing program that is based on large laboratories capable of assessing up to 9,200 tests/million inhabitants per day. The second, an ongoing surveillance testing program in nursing homes (also termed the “Protector of Fathers and Mothers” program) (https://corona.health.gov.il/en/magen-israel/). Third, an unparalleled, pro-active, national vaccination program (using the Pfizer-Biontech BNT162b2 vaccine), which reached a coverage of 80% for the first dose in the elderly, aged 60+ years, population within 38 days.^9^ We aimed to explore the transmission dynamics of the B.1.1.7 variant and to estimate the success of the abovementioned national operations to mitigate the risk in the general population and in the elderly. To that end, we analyzed primary data of 292,268 RT-PCR samples that were collected from December 6, 2020, until February 10, 2021 (Table 1). This timely data provides an ideal setting to understand the dynamics of the dominant B.1.1.7 variant in light of disease surveillance and vaccination programs.

**Table 1:** Cohort features. Demographic characteristics and disease features of the RT-PCR cohort

Characteristic	No. (%)
Total specimens	292,268 (100)
Total positive to COVID-19	12,891 (4.41)

**Sex**	

Female	95,945 (32.83)
Male	103,825 (35.52)
Unknown	92,498 (31.65)

**Specimen source**	

Community	91,975 (31.47)
Nursing homes	200,293 (68.53)

**Age group, years**	

0–19	71,519 (24.47)
20–59	161,192 (55.15)
60+	59,313 (20.30)
Unknown	244 (0.08)

## Results

### The B.1.1.7 is 45% more transmissible than the wild-type strain in Israel

**Figure 1:**
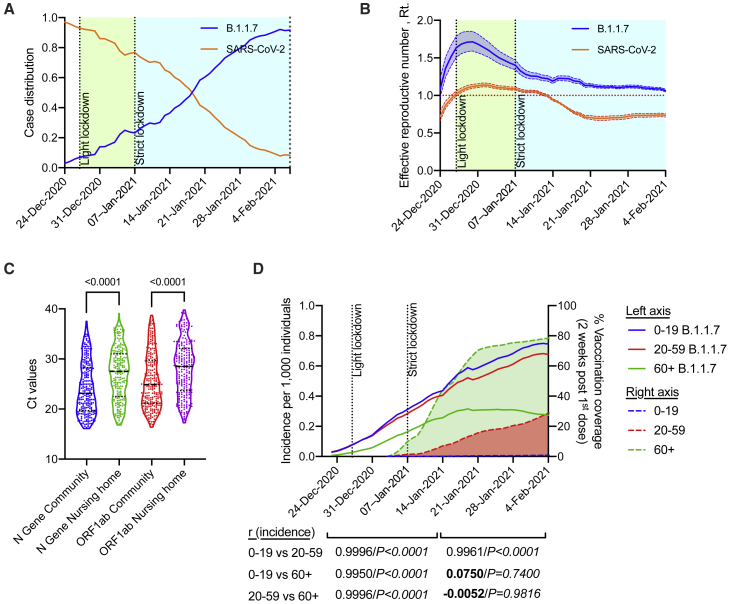
The emergence of variant B.1.1.7 in Israel (A) Daily new-case distribution between wild-type (SARS-CoV-2) and variant B.1.1.7 over time. Timing of lockdowns is indicated. (B) Effective reproduction number (*Rt*) of wild-type (SARS-CoV-2) and variant B.1.1.7 with 95% credible intervals over time. The effective reproduction number of variant B.1.1.7 was 1.45 (1.20–1.60) times higher than that of the wild-type. (C) The Ct value distribution (presented as a violin plot) for the N gene and the ORF1ab gene among infected individuals older than 60 years at nursing homes versus infected individuals older than 60 years in the general community. Data were calculated using GraphPad Prism 9; the black, dotted line represents the calculated median and quartile values. Statistical analysis was performed using a t test; p values are shown. (D) Incidence of viral infection with variant B.1.1.7 per 1,000 individuals, as stratified by age (left axis). Cumulative vaccination coverage 2 weeks after first dose per age group is shown (right axis). In (A), (B), and (D), data are from the analysis of 292,268 individual samples. In (C), each dot represents a different sample (no biological or technical replicates were performed). Data were calculated using GraphPad Prism 9; correlation analysis was performed using a Pearson correlation coefficient test (two-tailed, 95% confidence); r values are shown.

The B.1.1.7 variant possesses a deletion of two amino acids at positions 69–70 (Δ69–70), which is associated with the inability of the Thermo Fisher TaqPath COVID-19 assay probe to detect the Spike gene (S gene) (https://www.thermofisher.com/blog/behindthebench/thermo-fishers-covid-19-tests-designed-with-virus-mutations-in-mind/). Indeed, S-gene target failures (SGTFs) were recently shown to be primarily due to the new variant,^10^ and molecular studies have used this “Flat S” phenomenon to assess the transmission data of the B.1.1.7 variant.^10^ On the other end, the S gene of the other variants, for example, the B.1.351 and the P.2 (for updated list of the main SARS-CoV-2, see https://www.cdc.gov/coronavirus/2019-ncov/cases-updates/variant-surveillance/variant-info.html), which do not possess the Δ69–70, will be amplified. Thus, SGTFs will not be observed in those variants. To obtain SGTF data, RNA was extracted from nasopharyngeal swabs using a nucleic acid extraction automated station (Biomek i7) by magnetic bead separation, followed by one-step RT-PCR (Thermo-Fisher TaqPath COVID-19 assay kit). Using data from ∼300,000 individual tests (Tel Aviv University Ethics approval no. 0002746-2), which were collected from Israeli nursing houses and from random “Drive and Check” SARS-CoV-2 test complexes, we now describe the transmission kinetics of the B.1.1.7 variant in the Israeli population. Analyzing data that were obtained from December 6, 2020, until February 10 2021, enabled us to monitor the spread of B.1.1.7 variant and assess the impact of Israeli programs to reduce transmission. Until December 22, 2020, the B.1.1.7 variant was undetectable within the pool of positive cases in Israel (Figure 1A). Strikingly, within a period of 3.5 weeks, the B.1.1.7 variant outcompeted the previous wild-type, non-N501Y, dominant SARS-CoV-2 strain in Israel and became the dominant strain (by January 19, 2021; Figure 1A). Within 6 weeks, the B.1.1.7 variant was identified in more than 90% of positive tests (February 4, 2021; Figure 1A). The B.1.1.7 variant was 1.45 more transmissible than the wild-type strain (95% confidence interval [CI]: 1.20–1.60). This is within the range of previous estimates, which suggested a 1.56 increase in England.^10^

### The effective reproduction number of B.1.1.7

Next, we calculated the effective reproduction number *R*_*t*_, which is the expected number of new infections at time *t*, caused by an infectious individual. To evaluate the daily *R*_*t*_, we used the methodology previously introduced by Cori et al.^11^ This data-driven approach suggests that *R*_*t*_ is the ratio between *I*_*t*_, the number of new infections generated at day *t* and $\overset{t-1}{\underset{\tau =s}{\sum }}I_{t-\tau }w_{\tau }$, the sum of infection incidence up to time step *t* − 1, weighted by the infectivity function *w*_τ_. For *w*_τ_, we considered a 10-day, truncated γ-distribution, with a mean of 3.96 days (95% CI: 3.53–4.39 days) and an SD 4.75 days (4.46–5.07 days).^12^ We used our data to evaluate the reproductive number for both the SARS-COV-2 wild-type and the B.1.1.7 variant. For an accurate representation of Israel, we scaled our data with the age-stratified incidence data published daily by the Israeli Ministry of Health (MOH) (Database: https://datadashboard.health.gov.il/COVID-19/). Finally, we shifted the data by 3.5 days, which corresponded to the average time between exposure until obtaining the test outcome. Before the impact of the national lockdown, the *R*_*t*_ of the B.1.1.7 variant was as high as 1.71 (95% CI: 1.59–1.85) compared with 1.12 (95% CI: 1.10–1.15) observed for the wild-type (Figure 1B; December 30, 2021).

### Random surveillance testing in nursing homes reduces viral load

The rapid transmission rate of the B.1.1.7 variant prompted us to assess further whether the routine surveillance testing program in nursing homes was capable of early viral detection. Thus, we compared the Ct threshold values in 60+-year age groups in the general community versus in nursing homes. The Ct threshold values were used as predictive markers for viral load, in which, a higher Ct indicates lower mucosal viral titers. This analysis revealed significantly lower Ct values, which were not time-dependent, in both viral tested genes, in the community in comparison with nursing homes with matching age groups (t test, p < 0.001) (Figure 1C). The higher Ct values in nursing homes, which represent a lower viral load, are likely due to the ongoing surveillance program in nursing homes.

### Prioritized vaccination programs reduce viral transmission in the elderly

Increased viral transmission may have substantial implications on the ability to protect SARS-CoV-2-susceptible populations. Thus, we aimed to examine whether preventative policies, such as vaccination programs and focused protection interventions, can provid protection against the highly transmitted B.1.1.7 variant. Assessment of the distribution of the B.1.1.7 variant in different age groups revealed a clear increase in the numbers of B.1.1.7^+^ individuals from age groups 0–19 and 20–59 years. Although a similar increase was observed in the 60+-years aged group through January 13, 2021, the incidence of B.1.1.7^+^ individuals aged 60+ years plateaued and, subsequently, declined (Figure 1D). In support of this, Pearson correlation analysis of the rise in incidence among the different age groups revealed that between December 24, 2020, and January 13, 2021, the rise in the incidence of B.1.1.7 highly correlated among all age groups (r > 0.99, p < 0.0001; Figures 1D and S1A). It should be noted that during the first period (i.e., until January 13, 2021), the 0–19 age group linear distribution did not pass the D’Agostino-Pearson normality distribution test (Figure S1C; related to Figure 1D). However, after January 14, 2021, after which, all age groups showed normality distributions (Figure S1D; related to Figure 1D), a striking decline was observed in the correlation coefficient only in the 60+ years age group (r = 0.075, p = 0.74 and −0.005, p = 0.98; 60+ versus 0–19 or 20–59 years age, respectively; Figures 1D and S1B). This phenomenon was associated with the fact that, by that time point, 50% of the 60+ aged population was 2 weeks beyond the first dose of vaccination. This comparative age-based analysis demonstrated that transmission and spread of the B.1.1.7 variant among most of the population at risk (as define by being older than the age of 60 years) was significantly reduced in comparison with other age groups. Of even greater interest, we show that, although the transmission of B.1.1.7 continued to rise dramatically in both 0–19-year and 20–59-year age populations with similar kinetics, the rise in the 60+ population was completely halted.

## Discussion

Since the emergence of the SARS-CoV-2 pandemic, several variants have been identified, including the B.1.1.7 variant, associated with increased infectivity.^3^, ^4^, ^5^ Therefore, analysis of the dynamics of the spread of the B.1.1.7 variant is urgent and of great importance. Using SGTF data from more than 292,000 samples, we demonstrate that the B.1.1.7 variant is 45% more transmissible than the wild-type strain in Israel. We further demonstrate that active surveillance programs in nursing homes markedly reduced the transmission of B.1.1.7. Finally, we report that the prioritized vaccination program in Israel, which initially focused on vaccination of the elderly population, rapidly prevented B.1.1.7-associated infections in the elderly. Collectively our data show that proactive surveillance programs, combined with prioritized vaccination are achievable and can lead to decreased transmission, severe illness, and subsequent death.

Our analysis demonstrates that the B.1.1.7 variant was 1.45 more transmissible than the wild-type strain. These data are within the range of previous estimates, which suggested a 1.56 increase in England.^10^ Notably, because it is impossible to sequence all of our tested cases, there is a remote possibility that our data do not accurately reflect the spread of the B.1.1.7 variant in Israel. Nevertheless, that is unlikely because, in further support of our SGTF data, between January 1, 2021, and January 3, 2021, the MOH used random sequencing, and 27 of 190 samples were found to be positive for the B.1.1.7. (https://www.gov.il/en/Departments/news/01012021-01); https://www.gov.il/en/Departments/news/03012021-03). These data are in line with our SGTF data that demonstrated that the prevalence of the B.1.1.7 variant was approximately 16% during those dates.

Given the high transmission rate of the B.1.1.7 variant, we aimed to determine whether frequent and routine monitoring and surveillance programs of nursing homes in Israel were capable of early viral detection. In this program, nursing homes in Israel are tested approximately every 3 days, regardless of any concern of infection. By contrast, in the community, most of the tested population is either symptomatic or was in contact with confirmed COVID-19 patients. Our analyses revealed that the Ct values in nursing homes were higher than those in the matching-aged population. Because increased viral load drives transmission,^13^ our data underscore the important role of random surveillance testing in nursing homes and other high-risk communities.

Protection of populations that are susceptible to developing a severe disease because of SARS-CoV-2 infection is critical, especially given the emergence of variants, such as B.1.1.7, which display increased transmission. Specifically, although the efficacy of the Pfizer-Biontech vaccine has been demonstrated in clinical trials,^14^ data from real-world usage has not been reported against the B.1.1.7 variant. This issue is of great importance given the ongoing global vaccination programs and the spread of B.1.1.7 in additional countries.

Our data demonstrate a clear containment of the B.1.1.7 variant in the 60+ year age group after January 14, 2021, by the Pfizer-Biontech vaccine. Importantly, we observed a sharp decline in cases when ∼50% of the elderly population was 2 weeks beyond their first vaccination dose and at a time point during which the B.1.1.7 variant gained transmission dominance. In support of this finding, it was suggested that, after the first vaccination dose, more than 70% of patients develop neutralization antibodies,^15^ and the vaccine efficiency can reach 85%.^16^ These data suggest that the decline in viral transmission in the elderly population occurred because the vaccine elicited rapid and durable protection, even toward the B.1.1.7 variant. This interpretation is supported by the relative rise in B.1.1.7 transmission in ages 0–59 years, and the fact that, except for the vaccination regime, no other intervention measures were introduced in that age group at that time. Despite these data, the efficiency of the vaccine should be monitored over time to conclude that long protection is achievable.

Our results are among the first to report real-world data of vaccination efficacy in a large community cohort. We show that, despite increased infectivity and transmission of the B.1.1.7 variant, the Pfizer-Biontech vaccine (when ∼50% of the designated population are 2 weeks beyond their first vaccination dose) was capable of reducing its transmission. Furthermore, additional pro-active surveillance programs of populations at risk, such as those found in nursing homes, were capable of early detection, which likely enabled containment of further viral spread within this housing community. Thus, pro-active protection programs, such as routine surveillance and monitoring of populations at risk, combined with prioritized vaccination, are achievable and will reduce severe illness and subsequent death.

### Limitations of study

In our study, we monitor the dynamics of the spread of the B.1.1.7 SARS-CoV-2 variant in Israel. One possible limitation in our study is that we mostly rely on SGTF data and did not sequence the analyzed samples. Nonetheless, our SGTF data highly correlates with published reports regarding the spread of the B.1.1.7 variant in Israel by the Israeli MOH and health care providers in Israel (e.g., Clalit Health and Maccabi).

We demonstrate that the spread of the B.1.1.7 variant was halted in the age group of those 60+ in comparison with those of ages 0–59 years. Our interpretation of this data is that this phenomenon is due to the prioritized vaccination program. Nonetheless, we cannot exclude the possibility that this effect may be influenced by distinct behavioral differences among the different groups, such as social distancing and hygiene. In addition, we show that viral load was consistently lower in samples that were obtained from nursing homes compared with the general population. These data are attributed to the fact that nursing homes in Israel are frequently tested, regardless of disease symptoms. In contrast, community testing is primarily conducted in symptomatic or individuals suspected of infection. This interpretation bears the limitation that we do not control for prior infections in a given nursing home, which may result in natural immunity or in different behavior patterns, such as social distancing and personal hygiene. Nevertheless, the finding that there is no difference in the spread of the B.1.1.7 variant among the different age groups until the vaccination program was applied suggests otherwise. In fact, these data indicate that the marked difference is due to the impact of vaccination, which prioritized and targeted the 60+ age group.

## STAR★Methods

### Key resources table

**Table:** 

REAGENT or RESOURCE	SOURCE	IDENTIFIER
**Biological samples**		

292,268 Nasopharyngeal swabs	ELECTRA-TAU Laboratory	N/A

**Chemicals, peptides, and recombinant proteins**		

Ethanol	Biolabls. Israel	Cat number: 525052100
molecular grade water	Biolabls. Israel	Cat number: 232121232300
TaqPath™ 1-step Multiplex Master Mix No ROX	Applid Bioscience,	Cat number: A28523
MS2 Control phage	Applied Biosciences	Cat number: 100092698

**Critical commercial assays**		

MagMAX Viral/Pathogen Nucleic Acid Isolation Kit	Applied Bioscience	Cat number: A42352
lysis buffer	Beckman Coulter,	Cat number: C42153
magnetic beads	RNAdvance Viral Bind-VBE	Cat number: C58702

**Software and algorithms**		

GraphPad Prism 9 for macOS, version 9.0.1(128)	https://www.graphpad.com/scientific-software/prism/	SCR_002798

**Other**		

Israeli Ministry of Health daily COVID-19 cases stratified by age group	https://data.gov.il/dataset/covid-19	N/A
Israeli Ministry of Health daily vaccination coverage stratified by age group	https://data.gov.il/dataset/covid-19	N/A
QuantStudio 5 Real Time PCR System	Applied Bioscience	Cat number: AB-A28574
Biomek i7	Beckman Coulter	N/A
KingFisher 96 Flex	Thermo Fisher	N/A

### Resource availability

#### Lead contact

Further information and requests for resources and reagents should be directed to and will be fulfilled by the lead contact, Motti Gerlic (mgerlic@tauex.tau.ac.il).

#### Materials availability

This study did not generate new unique reagents

#### Data and code availability

The RT-PCR data supporting the current study are available from the Electra-TAU laboratory, but restrictions apply to the availability of these data, which were used under license for the current study and so are not publicly available. Data are, however, available from the authors upon reasonable request and with permission of the Electra-TAU laboratory. The age-stratified incidence and vaccination coverage daily data published by the Israeli Ministry of Health is available on https://datadashboard.health.gov.il/COVID-19/.

### Experimental model and subject details

The study was conducted under the Tel Aviv University Ethics approval number: 0002746-2.

Our data include RT-PCR tests from 292,268 individuals. The demographic characteristics of the cohort are shown in Table 1.

### Method details

#### Viral inactivation

Nasopharyngeal swabs were transported in 4°C to the Electra-TAU laboratory within 12 hours from the time of sampling. Samples were heat inactivated (50 minutes in 65°C followed by 15 minutes in room temperature).

#### RNA extraction

RNA was extracted from heat inactivated nasopharyngeal swabs using an integrated nucleic acid extraction automatic working station combining the Biomek i7 (Beckman Coulter) and KingFisher 96 Flex (Thermo Fisher) by magnetic bead separation (MagMAX Viral/Pathogen Nucleic Acid Isolation Kit, Applied Bioscience, Cat number: A42352) according to the manufacturer’s guidelines: 200 μL of heat inactivated sample was taken directly from the testing tube and added to a 96-well deep well plate (Thermo Scientific, Cat number: 95040450) containing 150 μL of lysis buffer (Beckman Coulter, Cat number: C42153), 350 μL of magnetic beads (RNAdvance Viral Bind-VBE, Cat number: C58702) and 10 μL of internal control (MS2 Control phage, Applied Biosciences, Cat number: 100092698). Thereafter, the plate was automatically transferred to KingFisher Flex (Thermo Fisher Scientific, Cat number: 5400610) incubated for 10 minutes to allow the binding of the magnetic beads to the RNA. Subsequently, the samples were washed twice with 80% ethanol (Biolabls. Israel, Cat number: 525052100) and eluted in molecular grade water (Biolabls. Israel, Cat number: 232121232300).

#### RT-PCR

Extracted RNA (17.5 μl) was transferred to 96 well PCR plate containing 7.5 μL of TaqPath™ 1-step Multiplex Master Mix No ROX (Applid Bioscience, Cat number: A28523). Followed by one-step RT-PCR (Thermo-Fisher TaqPath COVID-19 assay kit). Thereafter, the plate sealed with MicroAmp clear adhesive strip (Applied Bioscience, Cat number: 4306311). The plate was loaded onto a QuantStudio 5 Real Time PCR System (Applied Bioscience, Cat number: AB-A28574) and the following amplification program was used:

25°C for 2 minutes, X1 cycle
53°C for 10 minutes, X1 cycle
95°C for 2 minutes, X1 cycle
95°C for 3 s, followed by 60°C for 30 s, X40 cycles

Ct threshold values were preset using the following values/parameters: MS2- 15,000; by cycle 37; S gene- 20,000 by cycle 37; Orf1ab- 20,000 by cycle 37; N gene- 20,000 by cycle 37. Samples that passed the Threshold is a Ct value > 37 were re-tested or considered weak positive.

### Quantification and statistical analysis

Data were calculated using GraphPad Prism 9 for macOS, version 9.0.1(128).

#### Effective reproduction number, R_t_

To evaluate the daily $R_{t}$, we used the methodology previously introduced by Cori et al.^11^ This data-driven approach suggests that $R_{t}$ is the ratio between $I_{t}$, the number of new infections generated at day t and $\overset{t-1}{\underset{\tau =s}{\sum }}I_{t-\tau }w_{\tau }$, the sum of infection incidence up to time step t − 1 weighted by the infectivity function $w_{\tau }$. For $w_{\tau }$, we considered a 10 day truncated gamma distribution with a mean of 3.96 days (95% CI: 3.53-4.39 days), an SD 4.75 days (4.46-5.07 days).^12^ We used our data to evaluate the reproductive number for both the SARS-COV-2 wild-type and the B.1.1.7 variant. For an accurate representation of Israel, we scaled our data with the age-stratified incidence data published daily by the Israeli MOH. Finally, we shifted the data by 3.5 days, which corresponded to the average time between exposure until obtaining the test outcome. Median R_t_ values and 95% credible intervals, presented in the main text, were computed by sampling 1,000 pairs of mean and SD values for the truncated gamma distribution.

To evaluate to what extent the B.1.1.7 variant is more transmissible than the wild-type, we calculated for each day the $\frac{R_{t}^{B.1.1.7}}{R_{t}^{wild-type}}-1$. 95% credible intervals represent by day are presented. Namely, the lower bound represents the day where the value of this ratio is at the 2.5% percentile, and the upper bound represents the day where the value of this ratio is at the 97.5% percentile.

#### Random surveillance testing in nursing homes

To compare the Ct values in nursing homes and the community, we matched for every individual from nursing homes in a 1:1 ratio, based on year of age, to an individual in the community. Altogether, each group contains 314 individuals who tested positive for COVID-19. Unequal variances t test was used to compare the mean Ct values of the two groups.

#### Prioritized vaccination programs in the elderly population

We divided the incidence data into two periods: December 24, 2020, and January 13, 2021, and following January 14, 2021. This division is based on the point in time where more than 50% of the 60+ aged population was two weeks post the 1^st^ dose of vaccination. For each pair of age groups: 0-19y, 20-59y, and 60+y, we evaluated the Pearson correlation between each groupd in each time frame using a Pearson correlation coefficient test (two-tailed, 95% confidence). Statistical analysis was performed using a t test.

## Supplemental information

Document S1. Figure S1

Document S2. Article plus supplemental information
