# Supplementary material for: BNT162b2 vaccination effectively prevents the rapid rise of SARS-CoV-2 variant B.1.1.7 in high-risk populations in Israel
Source: Cell Rep Med. 2021 Apr 18;2(5):100264. doi: 10.1016/j.xcrm.2021.100264 (PMC8053239; doi:10.1016/j.xcrm.2021.100264)
Supplement: Document S1. Figure S1 [file mmc1.pdf]

Cell Reports Medicine, Volume *2*

Supplemental information

BNT162b2 vaccination effectively prevents the rapid rise of SARS-CoV-2 variant B.1.1.7 in high-risk populations in Israel

Ariel Munitz, Matan Yechezkel, Yoav Dickstein, Dan Yamin, and Motti Gerlic

Supplemental Figure

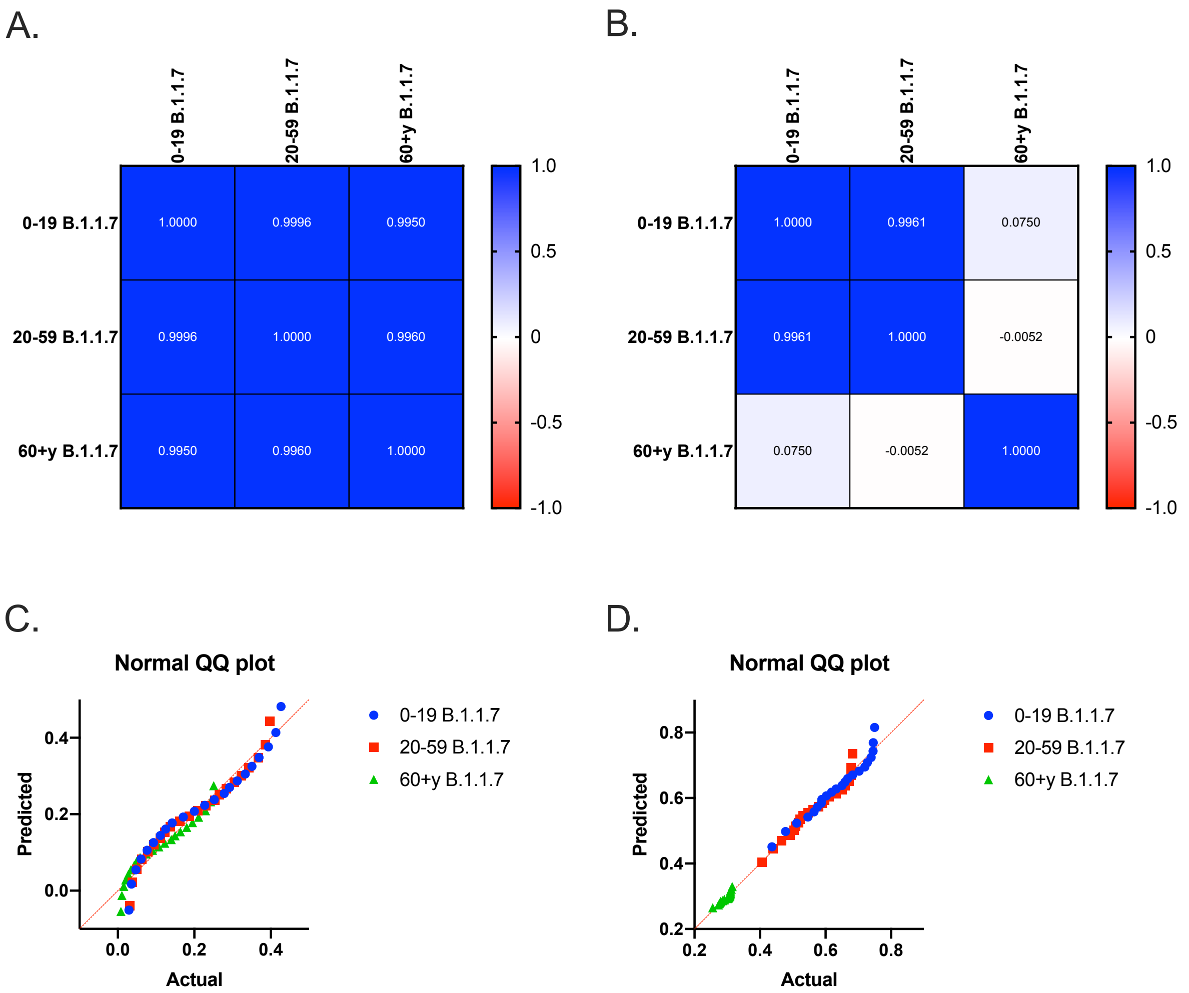

	0-19 B.1.1.7	20-59 B.1.1.7	60+y B.1.1.7
Test for normal distribution			
D'Agostino & Pearson test			
K2	6.329	5.036	5.070
P value	0.0422	0.0806	0.0793
Passed normality test (alpha=0.05)?	No	Yes	Yes

	0-19 B.1.1.7	20-59 B.1.1.7	60+y B.1.1.7
Test for normal distribution			
D'Agostino & Pearson test			
K2	1.286	1.669	5.217
P value	0.5257	0.4340	0.0736
Passed normality test (alpha=0.05)?	Yes	Yes	Yes

Figure S1 (Related to Figure 1D). Pearson correlation analysis (A, B) and Normal QQ plot and D'Agostino & Pearson normality distribution test (C, D.) related to Figure 1D. (A, C) December 24, 2020 until January 13, 2021; (B, D) January 13 until February 7, 2021. Data are from analysis of ~300,000 individual samples and were calculated using GraphPad Prism 9; Correlation analysis was performed using a Pearson correlation coefficient test (two-tailed, 95% confidence); r values are shown.
